# Le profil épidémiologique des complications maternelles de la césarienne au CHR EL Farabi Oujda

**DOI:** 10.11604/pamj.2017.27.108.10036

**Published:** 2017-06-12

**Authors:** Saad Benkirane, Hanane Saadi, Ahmed Mimouni

**Affiliations:** 1 Service de Gynécologie Obstétrique, CHU Mohammed, VI Oujda, Maroc

**Keywords:** Complications maternelles, césarienne, morbidité maternelles, mortalité maternelle, Maternal complications, cesarean section, maternal morbidity, maternal mortality

## Abstract

Au Maroc le taux de césariennes est passé de 2% en 1992 à 16% en 2011, cette augmentation est corrélée à une augmentation de la mortalité et de la morbidité per et post opératoire qui est de 19% dans notre série. Ce travail est le premier du genre à être effectué dans la région de l'oriental du Maroc dont l'objectif est d'analyser le profil épidémiologique détaillé des complications maternelles de la césarienne à partir de 2417 cas, à la maternité El farabi de Oujda, cette étude est une étude observationnelle descriptive rétrospective d'une série de 2416 patientes, ayant bénéficié d'une césarienne à la maternité de l'hôpital EL FARABI d'Oujda sur une période étalée entre le 1^er^ janvier 2011 et le 31 décembre 2013. 24464 accouchements étaient réalisés, dont 2416 cas par césarienne, soit un taux de 9.87%. La fréquence des complications liées à la césarienne était de 19.45%. Les complications postopératoires représentaient 63.6% des complications dominées par l'infection. Les complications hémorragiques représentaient 25.53% de toutes les complications. On a enregistré 4 cas de décès maternels. Si l'augmentation du taux de césariennes a contribué à l'amélioration du pronostic materno-fœtal, l'acte chirurgical en lui même n'est pas dénué de complications, ce qui nous incite à revoir ses indications pour une meilleure prise en charge de nos patientes.

## Abstract (en)

In Morocco cesarean section rate has increased from 2% in 1992 to 16% in 2011. This was associated with increased per- and postoperative mortality and morbidity, which was 19% in our case series. This study is the first of its kind to be conducted in the eastern region of Morocco and aims to analyze the comprehensive epidemiologic profile of maternal complications related to cesarean section on the basis of 2417 cases observed in the Maternity Department at the El Farabi Hospital, Oujda. We conducted an observational, descriptive, retrospective study of a series of 2416 patients undergoing cesarean section in the Maternity Department at the El Farabi Hospital, Oujda, over the period 1 January 2011-31 December 2013. Out of 24464 deliveries, 2416 were cesarean sections, reflecting a rate of 9.87%. The frequency of complications related to cesarean section was 19.45%. Postoperative complications accounted for 63.6% of the complications dominated by infection. Haemorrhagic complications accounted for 25.53% of all complications. 4 cases of maternal deaths were recorded. If the increased rate of cesarean sections has contributed to improve maternal-fetal prognosis, the surgical act itself is not complication-free, which leads us to review its indications for improved patient management.

## Introduction

La césarienne représente l'intervention la plus fréquente chez La femme, son taux n'a cessé d'augmenter au cours de ces trois dernières décennies et son incidence est passée de 5 à environ 25 voire plus de 50% dans certains pays. Au Maroc ce taux est passé de 2% en 1992 à < 16% en 2011 [1], devant cette situation nous nous retrouvons aujourd'hui fasse à une réelle problématique relative à la place raisonnable de cette intervention chirurgicale on obstétrique. Ainsi ce travail est le premier du genre à être effectué dans la région de l'oriental du Maroc dont l'objectif est d'analyser le profil épidémiologique des complications maternelle de la césarienne à partir de 2417 cas, à la maternité El farabi de Oujda, (Maroc).

## Méthodes

Notre étude est une étude observationnelles rétrospective menée sur une période de 3 ans, allant du 1er janvier 2012 au 31 décembre 2014, au service de gynécologie obstétrique du centre hospitalier régional (CHR) EL Farabi d'Oujda qui est un centre de niveau 2, il réalise près de 8000 accouchements par ans. Toutes nos patientes ont bénéficiers d'une délivrance dirigée, d'une antibioprophylaxie à base d'une amoxicilline protéger pendant 48h et d'une prévention de la maladie thromboembolique par un lever précoce au jour 1 du post-opératoire et par une héparinothérapie à bas poids moléculaire pendant 10 jours. Le recueil des données était fait à l'aide d'une fiche d'exploitation standardisée et l'analyse statistique des données à l'aide du logiciel SPSS version 20.

## Résultats

Durant cette période d'étude nous avons réalisé 22048 accouchements dont 2417 par césarienne (9.87%) et 470 ont présentés des complications per opératoires ou post opératoires.

**Étude globale:** L'âge moyen des césariennes compliquées été de 30.50±6.88 ans, avec des extrêmes d'âge compris entre 16 à 49 ans, 90% de nos patients habitaient dans un milieu urbain, et 14% étaient référer d'une maternité de niveau 1. Concernant la parité, 42.76 % étaient des primipares et 40.85% des paucipares. 26% des parturientes avaient un utérus cicatricielle et 81.7% était non suivies, la grossesse était á terme dans 79.15% des cas. L'acte opératoire était fait sous anesthésie général dans 73%, sous rachianesthésie dans 27%, et seulement 18% de nos césariennes étaient programmées, 82% des indications étaient posées en urgence et une hystérectomie d'hémostase était réalisée chez une 9 patiente.

**Morbidité maternelle:** Les complications maternelles de la césarienne sont représentées dans le (Tableau 1, Tableau 2). 36.6% étaient des complications peropératoires dominées par les hémorragies dans 120 cas soit 25.5% des complications, et qui représente 4.9% de toutes nos césariennes, dont 105 cas faisaient suite à des césariennes réalisées en urgence et 15 cas secondaires à des césariennes programmées. Les complications post opératoires représentaient 63.6% Elles étaient dominées par les infections de la paroi dans 35.7% suivi de l'endométrite du post-partum dans 10.6% des cas.

**Tableau 1:** Complications per-opératoires

	Nombre	Pourcentage par rapport aux complications	Pourcentage par rapport au total des césariennes
Hémorragie peropératoire	120	25,53	4,9
Fusion utérine	33	7,02	1,3
plaie vésicale	15	3,19	0,6
Plaie digestive	3	0,64	0,1
Complications anesthésique	0	0	0
Total	171	36,38	7

**Tableau 2:** Complications post-opératoires

	Nombre	Pourcentage (%) par rapport aux complications	Pourcentage (%) par rapport au totale des césariennes
Infection de la paroi	168	35,7	6,9
Endométrite du post-partum	50	10,64	2
Infection urinaire	35	7,4	1,4
Hémorragie du post-partum	30	6,38	1,2
Complications thromboemboliques	10	2,13	0,41
Hématome de la paroi	2	0,43	0,08
Eventration /éviscération	2	0,43	0,08
Pelvi péritonite	1	0,21	0,04

**Mortalité maternelle:** Dans notre série on a déploré quatre cas de décès maternels soit 0.16% des césariennes dont deux cas été suite á une hémorragie du post-partum, un cas pour choc septique et un cas pour suspicion d'embolie amniotique.

## Discussion

Ce travail sur le profil épidémiologique des complications de la césarienne est le premier du genre à être effectuée dans la région de l'oriental du Maroc La morbidité maternelle à court terme dans notre série était de 19% ce qui est proche des de données de la littérature qui ont été rapporter par Häger et al en Norvège (21.4%) en 2004, par Mariko et al en 2007 au Mali (22.7%) [2] et ceux retrouvé à l'Hôpital Central de Yaoundé en 2012 (20.11%), ces taux sont beaucoup plus élevés que ceux rapporter lors de l'accouchement par voie basse [3].

**Complications peropératoires:** Dans notre série 36.38 % des complications étaient des complications peropératoires ce qui représente 7% de nos césariennes, L'hémorragie peropératoire était la complication la plus fréquente, elle concernait 4.9% de nos patientes ce qui est légèrement inferieure au données de la littérature de 7.1 % [4]. Cette incidence est diversement appréciée selon la définition adoptée et leur moment de survenue. Elle a été causée essentiellement par l'inertie utérine, suivie par les lésions des pédicules utérins. Le recours à l'hystérectomie d'hémostase n'est envisagé qu'après échec des mesures conservatrices ou d'emblée en cas d'accident gravissime Sa fréquence dans notre série est de 0.37%, proche de celle retrouvée par Lau (0.32%) [5]. Si ces complications hémorragiques surviennent souvent en peropératoire, elles peuvent aussi survenir en postopératoire nécessitant une prolongation de l'hospitalisation, le recours à des transfusions sanguines voire à une reprise chirurgicale; Van Ham [6] rapporte que 4% des patientes avaient présenté un saignement postopératoire variant entre 1 000 et 1 500 ml, et 3.5 % avaient présentées des hématomes de la paroi. Dans notre série, le taux fusion utérine était de 1.4 % des césariennes, ce dernier était plus élevé que celui rapporter par Abbassi et al et par Bouamama qui trouvaient respectivement 0,2 [7] et 0.62% [8]. Les plaies digestives ainsi que les plaies vésicales sont comptées parmi les complications per opératoires les moins fréquentes au cours des césariennes. A travers une revu de littérature, les plaies vésicales représentent 1.4% [8] des complications tandis que les plaies digestives demeurent exceptionnelles. Dans notre étude, 15 patientes ont présenté une brèche vésicale soit un taux de 0.62% de l'ensemble des césarienne dont 12 cas à la suite d'une césarienne réalisée en urgence pour disproportion foetopelvienne. On a enregistrée également 3 cas de plaies digestives, soit un taux de 0.12%. Aucun cas de complications anesthésique n'a été enregistré au cours de notre étude

**Complications post-opératoires:** Dans notre série 63.6% des complications étaient des complications post opératoire, les complications infectieuses se positionnent au premier rang des complications post opératoire d'une césarienne. Les femmes qui accouchent par césarienne ont un risque 5 à 20 fois plus élevé de contracter une infection que les femmes qui accouchent par voie basse [9]. Elles entrainent une morbidité post natale importante, prolongent la durée d'hospitalisation et majorent significativement le coût financier. Leurs incidences varient de 7 à 25 % selon les auteurs et peuvent être très variables selon les critères retenus [10]. Dans notre étude, 10.4% des césariennes ont présentaient des complications infectieuses, et ses facteurs de risque sont représentés essentiellement par le caractère urgent de la césarienne et la rupture de la poche des eaux. Les complications infectieuses étaient dominées essentiellement par l'infection de la paroi suivie de l'endométrite. Les infections de paroi correspondent à une contamination de la cicatrice par des bactéries d'origine cutanée ou vaginale. Leur incidence varie de 0.4 à 9.8% avec une fréquence plus importante chez les patientes obèses [11], dans notre série nous avons trouver 6.9%. Il faut souligner que de nombreux travaux ont démontré que moins de la moitié des infections des sites opératoires sont diagnostiquées avant la sortie de la patiente de la Maternité, et que 40 à 80 % de ces infections surviennent après la sortie de la Maternité. L'incidence globale de ces complications est donc particulièrement sous-estimée si elles ne sont pas recherchées après la sortie de la patiente [12]. Concernant les endométrites leurs incidence est diversement appréciée par les auteurs. Elle est de 2% dans notre série sous antibioprophylaxie systématique de 48h, cette fréquence est faible par rapport à celle retrouvée dans une méta-analyse de la Cochrane en 2010 [13], l'incidence de l'endométrite du post-partum est de 1,5 % après une césarienne programmée avec antibioprophylaxie et 3.9% sans antibioprophylaxie. Dans notre étude, 35 patientes ont présenté une infection urinaire, soit un taux de 1.4% ce qui est certainement sous-estimée du fait de l'absence de sa recherche d'une manière systématique; Van Ham [6] a rapporté une fréquence de 3%. On a enregistré 10 cas de thrombophlébite du membre inférieur, soit un taux de 0.41% malgré la prévention systématique par le lever précoce et l'héparine de bas poids moléculaire pendant 10 jours, les facteurs de risque représentés essentiellement par: l'âge avancé, le non suivi des patientes et le caractère urgent de la césarienne Clement [14] rapporte une fréquence de 0.34 % et retrouve comme principaux facteurs de risque la multiparité, l'obésité, les antécédents thromboemboliques, l'alitement prolongé, les perturbations de l'hémostase et l'infection. On a enregistré également 2 cas d'éventrations soit un taux de 0.08%. Ces 2 cas ont été repris chirurgicalement, et dont les suites post opératoires étaient sans anomalies. Selon la littérature, c'est une complication rare, et elle est surtout liée à la technique opératoire utilisée. On a enregistré un cas de pelvipéritonite, soit un taux de 0.21%.

**Mortalité maternelle:** De 1983 à 1992, Hickl [15] a analysé la mortalité globale et la mortalité spécifique (celle proprement liée au mode d'accouchement et non au terrain): la mortalité maternelle globale après césarienne et après un accouchement par voie basse était, respectivement, de 0.44‰ et 0.04‰, et la mortalité spécifique après césarienne était 7 fois supérieure à celle après voie basse (0.190‰ versus 0.027‰). De même, Lilford [16], dans une étude réalisée en Afrique du Sud entre 1975 et 1986, rapporte une mortalité spécifique 5 fois plus élevée après césarienne comparativement à la voie basse. Pendant la période étudiée, On a effectué 22048 accouchements. Nous avons déploré 4 décès pour 2417 césariennes, soit un taux de mortalité par césarienne de 0.16‰

## Conclusion

Si l'augmentation du taux de césariennes a contribué à l'amélioration du pronostic materno-fœtal, l'acte chirurgical en lui même n'est pas dénué de risques. Les complications fréquentes hémorragiques per-opératoires, infectieuses et thromboemboliques, nous incitent à revoir nos indications de la césarienne.

### Etat des connaissances actuelle sur le sujet

- Le taux de complications maternelles de la césarienne dans la littérature est élevé au alentour de 20%;
- Les publications dans la littérature ont peu d'effectif.

### Contribution de notre étude à la connaissance

- Ce travail est le premier du genre à être effectué dans la région de l'oriental du Maroc dont l'objectif est d'analyser le profil épidémiologique détaillé des complications maternelles per opératoires et post opératoires de la césarienne;
- ce travail a inclus un effectif important de 2417 cas de césariennes.

## Conflits d’intérêts

les auteurs ne déclarent aucun conflit d'intérêts.
